# Core Self‐Reported Variables for Next‐Generation Colorectal Cancer Risk Prediction: An International Expert Consensus Study

**DOI:** 10.1002/cam4.72348

**Published:** 2026-10-22

**Authors:** Hanna Dubois, Eva Torbjörnsson, Jessica Norrbom, Alan Hubbard, Mirna Enskär, Rachael V. Phillips, Mark van der Laan, Johannes Blom

**Affiliations:** ^1^ Department of Clinical Science and Education Södersjukhuset Karolinska Institutet Stockholm Sweden; ^2^ Theme Cancer Karolinska University Hospital Stockholm Sweden; ^3^ Department of Perioperative and Intensive Care Södersjukhuset Stockholm Sweden; ^4^ Department of Physiology and Pharmacology Karolinska Institutet Stockholm Sweden; ^5^ Division of Biostatistics, School of Public Health University of California at Berkeley California USA; ^6^ Department of Surgery Södersjukhuset Stockholm Sweden

**Keywords:** colorectal cancer, delphi technique, expert consensus, machine learning, risk prediction, screening

## Abstract

**Background:**

Colorectal cancer remains a major global health burden, and although population‐based screening is beneficial, current programs have notable limitations. Risk‐stratified screening has been proposed, yet existing prediction models vary in performance and in the inclusion of predictors. Data‐adaptive modeling (machine learning) may strengthen risk prediction but depends on high‐quality input data. Expert consensus methods offer a structured way to identify clinically meaningful variables for future tools.

**Objective:**

To establish expert consensus on the selection of relevant variables and refinement of self‐reported questionnaire items for next‐generation colorectal cancer risk‐prediction tools.

**Methods:**

A modified Delphi study was conducted with seventeen internationally recognized experts. The process consisted of anonymized, email‐based rounds aimed at refining a questionnaire for colorectal cancer risk prediction. Across three iterative rounds, experts rated item relevance, proposed revisions, and identified missing domains while reviewing group‐level summaries of ratings and comments. Items not meeting predefined thresholds were removed, and validated lifestyle‐related items were incorporated. Consensus was defined as 15 of 17 experts rating an item within a similar three‐point range.

**Results:**

All seventeen experts completed all rounds. The final *I‐CRC‐RISK Questionnaire* comprises 23 items across seven categories: *Demography*, *Health Status, Family Health History, Dietary Patterns, Physical Activity, Smoking*, and *Alcohol Consumption*. Expert consensus was reached for 19 items.

**Conclusion:**

The *I*‐*CRC*‐*RISK Questionnaire* provides an expert‐informed set of self‐reported variables to support future risk prediction efforts. It offers a structured basis for further validation and psychometric testing before prospective data collection may be undertaken. This foundational work could enhance the predictive performance of both traditional and machine‐learning approaches by improving the consistency and relevance of input data.

## 1 Introduction

Colorectal cancer (CRC) is the third most diagnosed cancer worldwide, with about 1.9 million new cases and 930,000 deaths annually [1]. Incidence is rising among younger adults, increasing the need for effective prevention and early detection [2]. Population‐based screening aims to identify CRC at an early curable stage in asymptomatic individuals and can also prevent cancer by removing precancerous adenomas [3]. In the European context, CRC screening is widely implemented and remains a policy priority within the EU's organized and quality‐assured screening programs [4]. In most population‐based screening settings, a positive fecal occult blood test serves as the indication for subsequent colonoscopy. However, most positive stool tests are false positives, exposing participants to follow‐up procedures without direct benefit but with the risk of complications [5]. A more serious concern for individuals is the occurrence of so‐called interval cancers, i.e., CRC diagnosed outside the program between screening rounds (missed cancers), which further highlights limitations of current programs [6]. Screening methods, age limits, and intervals vary between programs and are, consequently, continuously being discussed [7]. Risk‐stratified screening has been proposed to reduce both over‐ and under‐screening [8, 9, 10, 11], and further research has been called for to develop and validate effective risk prediction tools [12, 13].

Systematic reviews demonstrate that although numerous CRC risk prediction models have been developed, their performance and methodological rigor remain inconsistent [14, 15]. Models vary substantially in the predictors they include, external validation, or evidence of clinical utility [14, 15, 16]. Despite their clear epidemiological relevance, lifestyle factors are incorporated unevenly across models, further limiting comparability [17]. This highlights the need for more robust, systematically developed prediction models grounded in clinically relevant and consistently defined predictors [15, 18].

Current risk prediction models predominantly use traditional statistical methods, e.g., Cox proportional hazards modeling or logistic regression. Only more recently developed ones rely on machine learning (ML) [19, 20, 21]. Despite their strengths, ML models often rely on complex and data‐driven variable selection that may not align with clinical priorities. In general, such approaches work only if the covariates used contain the most important predictors. In short, these methods are powerful at leveraging the information available in the covariates for risk prediction, but they can only work with the information available in the predictors. Available data, in patient charts or registries, do not always include all potentially relevant variables. Particularly, lifestyle variables in registries are largely missing and are often only available through prospective collection via self‐reported questionnaires. Any prediction modeling approach will perform better if more informative predictors are added to the datasets.

Expert consensus methods offer a transparent, systematic, and multidisciplinary approach to define input variables, enhancing both interpretability and clinical applicability of future risk‐prediction models. The Delphi method is a well‐established technique for iterative and systematic collection of expert opinion, particularly suited to complex topics where empirical evidence may be limited or emerging [22, 23]. As a foundational step, prior to psychometric validation of questionnaire items, a multidisciplinary expert panel could ensure that important predictors are not omitted and consider aspects such as feasibility, ethics, data availability, and patient acceptability, relying on their research and clinical experience.

The aim of this study was to establish expert consensus on the selection and curation of self‐reported variables that will inform the development of next‐generation CRC risk‐prediction tools.

## 2 Materials and Methods

### 2.1 Initial Questionnaire Draft—Item Generation

The development of the initial draft of a comprehensive risk‐prediction questionnaire started in 2023. Literature on predictors for colorectal cancer [24, 25, 26, 27, 28, 29, 30, 31, 32, 33, 34] was reviewed and informed the construction of the first draft. Particular attention was given to lifestyle‐related variables, such as dietary habits and physical activity, although these domains presented challenges in terms of operationalization and questionnaire design. In the first version, response options for physical activity were adapted from indicator questions from the National Board of Health and Welfare, Sweden [35]. The first questionnaire draft consisted of a total of 30 items in categories: *Demography, Health Status, Family Health History, Dietary Patterns* (featuring two alternative response formats), *Physical Activity, Smoking*, and *Alcohol Consumption*.

Using the initial questionnaire draft as a foundation, a Modified Delphi study was conducted between September 2024 and May 2025, with the aim of identifying relevant self‐reported variables for CRC risk prediction and refining the questionnaire items used to measure them.

### 2.2 Participants

A purposive sampling strategy was used to recruit experts based on their recognized CRC expertise and experience in primary and secondary cancer prevention. First, candidates were identified based on recommendations from the International Agency for Research on Cancer (IARC)—the World Health Organization (WHO) [36]. Additional experts were recruited through snowball sampling [37], where the initially approached experts suggested suitable candidates. A total of 27 experts were approached, of which 17 agreed to participate in the study. Four experts declined to participate (three due to time constraints and one due to parental leave), and six did not respond despite a reminder sent 1 month after the initial invitation.

The process to select experts and to determine the size of the expert group adhered to the Delphi methodology, which typically involves 15–20 experts to ensure both depth and breadth of opinion [22, 23].

The expert group included eight female and nine male participants from Australia, Canada, Italy, the Netherlands, Saudi Arabia, Spain, Sweden, Switzerland, the United Kingdom, and the United States. A complete list of the experts and their credentials is provided in Supporting Information S1.

### 2.3 Data Collection

Three consecutive email‐based Delphi rounds were conducted, and up to three reminders were sent out for each round. As part of the Delphi methodology, anonymity between expert group members was maintained throughout the process. An overview of the Delphi process is provided in Figure 1.

**FIGURE 1:**
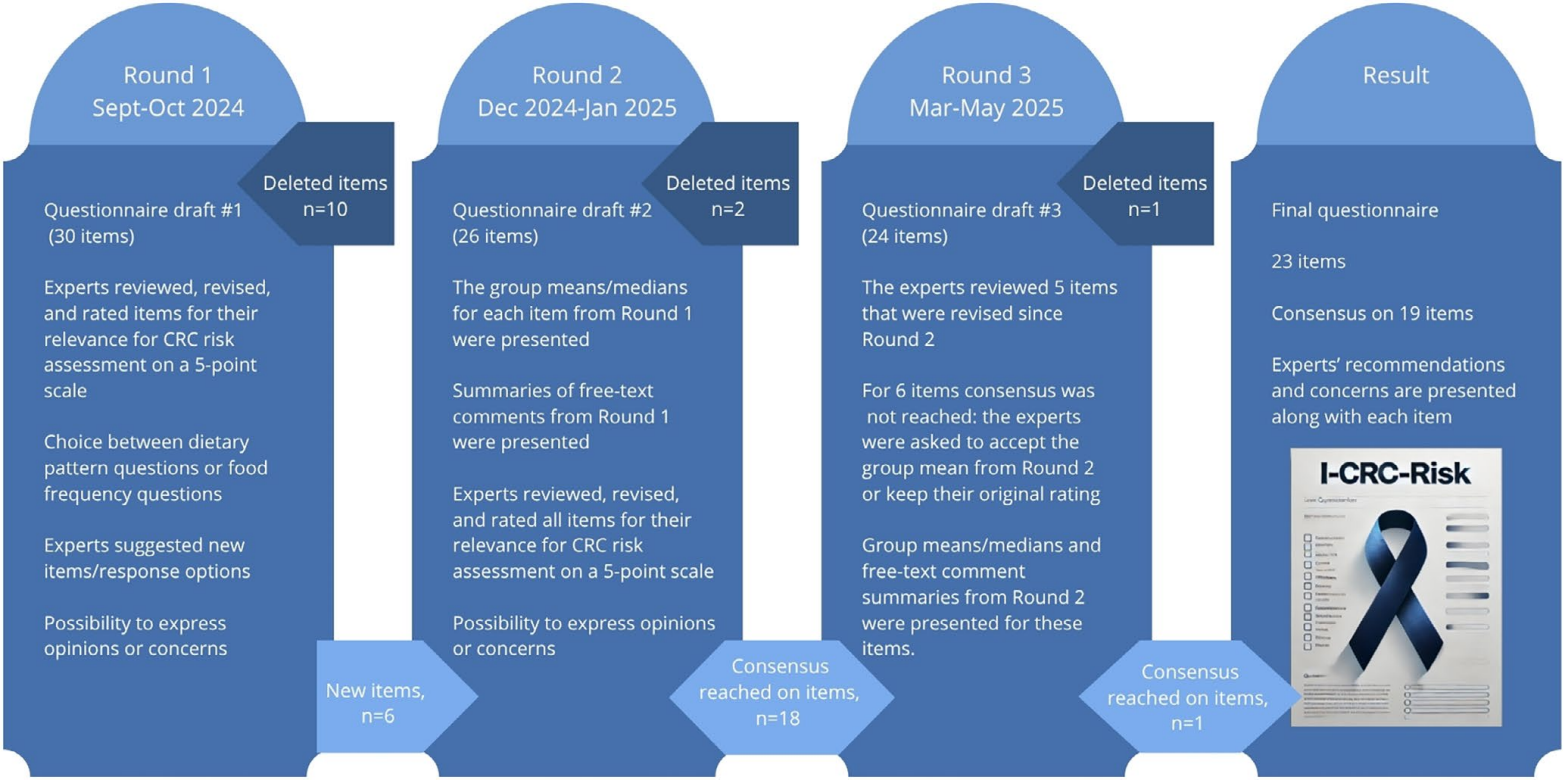
Overview of the Delphi process to develop and refine the I‐CRC‐Risk Questionnaire.

### 2.4 Round 1

The expert group was provided with an initial draft of the questionnaire, consisting of 30 items, formatted as a spreadsheet in Excel. They were instructed to evaluate the content for clarity, consistency, and potential redundancies, as well as to identify any missing domains. Experts were encouraged to revise existing categories and items, suggest new ones, and assess the relevance of each item for CRC risk prediction using a 5‐point Likert scale (1 = not relevant at all, 5 = highly relevant).

For items related to dietary patterns, the experts were asked to indicate a preference between general dietary pattern questions and food frequency questions. To support decisions regarding item retention, experts were given the opportunity to explicitly state whether each item should be retained or removed from the questionnaire. The dietary items were not rated in Round 1. The experts were encouraged to reach out if they needed support with the spreadsheet. Additional assistance was provided via email, on two occasions, in person, and over a video‐conference call.

### 2.5 Round 1 Analysis and Revisions

Mean and median values of the ratings were calculated for each item (except for the dietary items). Items with a median rating of ≤ 3 were excluded from the questionnaire. New items suggested by the experts were incorporated into the revised questionnaire draft. Additionally, several experts highlighted in their free‐text comments the need for validated questions concerning food frequency and physical activity. Consequently, food frequency items were incorporated into the questionnaire from the LifeCRC score, originally developed by Aleksandrova et al. in 2021 [19], and modified and externally validated by Brenne et al. in 2024 [38]. The Saltin‐Grimby Physical Activity Level Scale [39] was included as it is a simple, well‐validated, widely used assessment tool for evaluating physical activity level in epidemiological research. It was chosen because it captures habitual, long‐term physical activity across both occupational and leisure domains [40].

The free‐text comments were compiled and reviewed by the research team. Where feasible, expert suggestions were included in the subsequent round. However, due to occasional discrepancies in expert opinions, particularly regarding the level of detail in response options, it was not always possible to accommodate all viewpoints in the revised version. In such cases, the most commonly expressed perspectives were prioritized. Divergent opinions were documented and presented alongside the revised items for transparency.

### 2.6 Round 2

In the second round, the experts revised the new draft, now a 26‐item questionnaire. For the items rated in Round 1, the experts were presented with the group mean and median values, and a summary of the group's free‐text comments. They were instructed to rate all items as well as state their comments or concerns.

### 2.7 Round 2 Analysis and Revisions

Mean and median values were calculated for each item rating, which further guided the retention of items for the final round; i.e., items with a median rating of ≤ 3 were excluded. Consensus on the mean rating of an item was defined as when 15 of the 17 experts' ratings of an item fell within a range of three consecutive values [41]. As there is no universally accepted definition of consensus in Delphi studies and substantial variation exists in the thresholds used [41], we applied a stringent consensus criterion of ≥ 15/17 experts (88% agreement). Given the relatively small expert panel, this threshold was considered to represent a high level of consensus. In addition, ratings were required to fall within a 3‐point range on the 5‐point scale, allowing for minor variation in individual responses while ensuring substantial agreement among experts.

The free‐text comments were compiled and reviewed by the research team. Small revisions were made based on the expert comments.

### 2.8 Round 3

The number of rounds in Delphi studies typically ranges from two to four [42]. After Round 2, considering the quality of the feedback and the degree of convergence in experts' ratings, it was concluded that a third and final round would be sufficient. The experts were informed that the third round would be the last and were only presented with items that had been revised after Round 2 (*n* = 5) and with those where consensus was not obtained (*n* = 6). The experts were asked to accept or reject the revisions and to either accept the group means or keep their rating from Round 2.

### 2.9 Round 3 Analysis and Revisions

As all proposed revisions were accepted by the expert panel, no further modifications were required. Consensus was considered to be achieved when more than 15 experts approved of the group mean from Round 2; in such cases, the Round 2 mean was retained. For items where more than two experts kept their original ratings instead of aligning with the group average, a revised mean was calculated. In this calculation, ratings from experts who agreed with the group average were replaced by that value, while the original ratings from those who did not were retained.

### 2.10 Ethical Considerations

The Swedish Ethics Review Authority approved the study (Dnr 2024–04407‐01). The invited experts participated in their professional capacity as subject‐matter experts and contributed to the development of the questionnaire through their expert judgments and feedback. All experts consented to the publication of their names and affiliations to ensure transparency regarding the expertise represented in the panel. We therefore considered their role to extend beyond that of traditional anonymous research participants and to include collaboration in the instrument‐development process.

## 3 Results

Three consecutive Delphi rounds were carried out, with the participation of 17 experts, without any loss of participants.

The final version, the ‘*I‐CRC‐RISK Questionnaire’*, contains 23 items, divided into seven categories: *Demography*, *Health Status*, *Family Health History*, *Dietary Patterns*, *Physical Activity*, *Smoking*, and *Alcohol Consumption*. The development of the *Dietary Patterns* items and *Physical Activity* items was guided by two validated questionnaires, i.e., the LifeCRC Questionnaire [38] and the Saltin‐Grimby Physical Activity Level Scale [39].

Expert consensus on the relevance for risk prediction was reached for 19 of the 23 items included in the final questionnaire. On a 5‐point Likert scale, the expert ratings on the included items in the *I‐CRC‐RISK Questionnaire* had a mean score of 4.26 (SD = 0.50), a median of 4.29 (IQR 3.97–4.68), and a range of 3.31–5.00.

Overall, items representing established demographic, hereditary, and lifestyle‐related risk factors received the highest ratings, i.e., *Sex*, *Age*, *History of CRC in the family*, *Known hereditary high‐risk predispositions in the Family*, *Smoking*, and *Alcohol Consumption*.

Four items failed to reach expert consensus: *Household Income*, *Dark Bread*, *Dairy Products*, and *Red Meat*. The ratings for these items reflected divergent expert opinions, with individual ratings falling both above and below the mean from Round 2.

For *Household Income*, expert comments highlighted concerns regarding the feasibility of data collection, particularly due to the sensitive nature of the question and the potential for missing responses. At the same time, several experts considered income to be a relevant indicator of socioeconomic status.

For the three dietary items that did not reach consensus (*Dark Bread*, *Dairy Products*, and *Red Meat*), the experts questioned whether the items adequately captured the intended dietary exposures. Additional concerns related to the state of evidence, cultural variation, respondent burden, and the balance between collecting detailed dietary information and maintaining simplicity and feasibility.

An additional item proposed by the expert panel was *Previous participation in CRC Screening or Previous Colonoscopy*. Although the experts considered this an important risk‐related variable, concerns were raised regarding the validity of self‐reported information, particularly details relating to premalignant lesions and previous screening findings.

The full *I‐CRC‐RISK Questionnaire* is presented in Table 1, including mean ratings. In Table 2, information on consensus as well as a summary of expert comments is presented. Items that were excluded from the final version are shown in Table 3. The full anonymized dataset from the three Delphi rounds is provided in Supporting Information S2, including ratings, revisions, and comments.

**TABLE 1:** The final *I‐CRC‐RISK questionnaire*, including items, response options, and their mean relevance for CRC risk prediction using a 5‐point Likert scale (1 = not relevant at all, 5 = highly relevant) rated by the expert panel.

Item number	Question	Response options	Mean relevance for CRC risk prediction (Likert scale1–5)
Demography			
1	Are you male or female? Please answer using your sex at birth.	Male Female	4,94
2	Age	Years	5
3	Household income (yearly)	Low Middle High	3,33^a^
Health status			
4	Previously diagnosed with diabetes?	Yes (Type I/II/? Duration of disease and treatment) No I don't know	4,12
5	Height and weight (to calculate Body Mass Index).	Height and weight units adapted to the country in which the questionnaire is being used. Body Mass Index (via integrated calculator in an electronic questionnaire)	4,47
6	Waist circumference	• Cm or inches (instructions on how to measure provided) • Unable to measure	3,94
7	Previously diagnosed with Inflammatory bowel disease, i.e., Crohn's disease or Ulcerative colitis? (Explanation provided, e.g., a chronic inflammatory condition through your gastrointestinal tract which has been diagnosed by a specialist—not to be confounded with irritable bowel syndrome)	• Yes (Crohn's disease of the colon: Years from diagnosis, medication, hospitalization, surgery) • Yes (Ulcerative colitis): Years from diagnosis, medication, hospitalization, surgery • No • I don't know	4,65
8	Do you use any of the following drugs? (If possible, specify for each drug: Daily use/intermittent use, dose, duration of treatment)	• Aspirin • Other non‐steroidal anti‐inflammatory drugs (e.g., ibuprofen, naproxen) • Metformin • Statins (e.g., atorvastatin, simvastatin, pravastatin) • GLP‐1 receptor agonist (include list of brands) • Calcium • Vitamin D • Female hormonal replacement drugs	4
9	Previous cancer diagnosis?	• Yes (CRC—year) • Yes (endometrial, ovarian, stomach, small intestine, urinary tract, biliary tract, brain, pancreatic, thyroid, liver—year) • Yes, treatment included radiotherapy to the abdomen (year) • No	4,53
10	Previous participation in CRC screening or previous colonoscopy?	Yes (year/s): • Fecal test, years/s, results (positive for blood/negative for blood) • Colonoscopy, years/s, findings: • None or benign lesions • Premalignant lesions, i.e., advanced adenoma/sessile serrated lesion • Cancer No/I don't know	4,65
11	History of any of the following surgery?	Yes (year/s): • Colectomy • Bariatric surgery • Cholecystectomy No/I don't know	3,31
Family health history			
12	History of CRC in the family:	Yes (age at diagnosis), number of family members with history of CRC: • First degree relative (parent, sibling, child) • Second degree relative (grandparents, grandchildren, aunts, uncles, nieces, and nephews) • Third degree relative (first cousin, great grandparents) No/I don't know	4,82
13	Known hereditary high‐risk predisposition syndrome in the family, e.g., Lynch syndrome, FAP, MAP?	• No/I don't know • Yes: In the family (I have been tested, and I don't have the genetic syndrome) • Yes: In the family, potentially also me (I have not been tested for the genetic syndrome) • Yes: I have been tested and positive for …. Which syndrome? • Hereditary non‐polyposis colorectal cancer syndrome, e.g., Lynch syndrome • Hereditary polyposis syndrome e.g., Familial adenomatous polyposis (FAP), MUTYH‐Associated Polyposis (MAP), Serrated polyposis syndrome	4,71
Dietary patterns			
14	Fruit and berries	“How often do you normally eat this type of food?” 0–3 times per month 1–3 times per week 4–6 times per week Once a day Two or more times per day	3,82
15	Vegetables	“How often do you normally eat this type of food?” 0–3 times per month 1–3 times per week 4–6 times per week Once a day Two or more times per day	4,12
16	Dark bread	“How often do you normally eat this type of food (wholemeal bread, rye bread, cereal bread, brown wheat bread)?” 0–3 times per month 1–3 times per week 4–6 times per week Once a day Two or more times a day	3,74^a^
17	Dairy products	“How often do you consume dairy products such as milk, yoghurt, cheese, milk‐based puddings?” Never < 1 servings/day 1–3 servings/day > 3 servings/day	3,58^a^
18	Processed meat	“How often do you normally eat this type of food (e.g., smoked sausage, cured, salted or smoked meat, such as ham, bacon, jerky)?” 0–3 times per month 1–3 times per week 4–6 times per week Once a day Two or more times per day	4,41
19	Red meat (livestock meat)	“How often do you normally eat this type of food (beef, veal, pork, lamb)?” 0–3 times per month 1–3 times per week 4–6 times per week Once a day Two or more times per day	4,03^a^
Physical activity			
20	Occupational physical activity I experience my physical work situation/everyday life as: 1. Mostly sedentary sitting with elements of mobile work 2. Alternating between sitting and walking during the day 3. Periodically physically demanding work 4. At times very physically demanding work	Age (Enter Level 1, 2, 3, or 4 during the various periods of your life.) 20–30 30–40 40–50 50–60 Over 60	4,06
21	Recreational physical activity How much do you move and exert yourself physically in your spare time? If your activity varies a lot between summer and winter, for example, try to take an average. 1. Mostly sedentary leisure time You mostly devote yourself to reading, TV/screen, computers, cinema, or other sedentary activities in your spare time. 2. Some light to moderate physical activity in your free time for at least 4 h per week: You walk, cycle, or move in any other way for at least 4 h a week. This also includes walking or cycling to and from work, as well as Sunday walks, gardening, fishing, table tennis, bowling. 3. Regular moderate to heavy physical activity and exercise for at least 2 to 3 h per week You engage in e.g., heavy gardening, running, swimming, exercise gymnastics, tennis, badminton, or similar activities (please note that it must be in average at least 2–3 h per week). 4. Regular hard physical training and/or competitive sports (high‐intensity activity) You devote yourself to hard training or competition in running, orienteering, skiing, swimming, football, handball, etc., several times a week.	Age (Enter Level 1, 2, 3, or 4 during the various periods of your life.) 20–30 30–40 40–50 50–60 Over 60	4,29
Smoking			
22	Do you smoke (tobacco) cigarettes?	• Yes (For how many years? Number of cigarettes per day?) • No, but I have smoked in the past (For how many years, how much? When did you quit?) • No, I have never smoked.	4,82
Alcohol consumption			
23	During a typical week, how much alcohol do you drink?	Units/week (a table of examples of units/sort of alcoholic beverage should be provided, preferably with pictures). How do your current drinking habits compare to your habits 5 years ago? 10 years ago? More/less/the same?	4,71

^a^ Consensus was not reached for this item within the expert panel regarding the mean relevance for CRC risk prediction.

**TABLE 2:** Items in the *I‐CRC‐RISK questionnaire*, expert ratings, consensus, and a summary of the comments from the Delphi rounds.

Item number	Item	Mean relevance for CRC risk prediction (Likert scale1–5)	*n*/*N* (%) agreement 2nd round	Consensus 2nd round	*n*/*N* (%) agreement 3rd round	Consensus 3rd round	Comments and considerations
1	Sex	4,94	17/17 (100%)	Yes			Experts considered that the question should capture sex assigned at birth, while gender identity might be of interest from a research perspective.
2	Age	5	17/17 (100%)	Yes			Often possible to automatically require in health records.
3	Household income	3,33	13/17 (76%)	No	14/17 (82%)	No	Proxy for socio‐economic status (SES): Income level should be defined according to local standards in the country or context where the questionnaire is used. Other options discussed as proxies for SES included education level (not rated higher than 3 by the group) and residential area. The expert group expressed concerns that the sensitive nature of this item may lead to reporting bias among respondents.
4	Diabetes	4,12	17/17 (100%)	Yes			The experts differed in opinion regarding the level of detail in the response options, mainly due to concerns with feasibility/reporting bias.
5	Body Mass Index	4,47	16/17 (94%)	Yes			In addition to BMI as a predictive measure, height was also mentioned as a reflection of the standard of living. The expert group raised concerns about reporting bias.
6	Waist circumference	3,94	15/17 (88%)	Yes			The experts raised concerns about feasibility/reporting bias.
7	Inflammatory Bowel Disease	4,65	17/17 (100%)	Yes			The expert group raised several concerns that should be taken into account regarding the difficulty of assessing the predictive value of different treatment regimens for CRC risk. Duration of disease was recognized as important; however, individuals who know the duration of their disease may be more likely to adhere to surveillance programs. As a result, undertreated individuals may not be identified through this question.
8	Drugs	4	16/17 (94%)	Yes			Aspirin (acetylsalicylic acid) was frequently mentioned as the most important drug to be included in the questionnaire, as it is considered a preventive factor. However, opinions regarding the predictive value of the other drugs in the list were divided.
9	Previous cancer diagnosis	4,53	17/17 (100%)	Yes			The relevance of different types of previous cancers was discussed within the group. History of CRC was considered the most important. The other cancers on the list were discussed for inclusion, as they may be associated with an unknown hereditary syndrome.
10	Previous participation in CRC screening or previous colonoscopy	4,65	17/17 (100%)	Yes			The experts discussed the feasibility of this item—specifically, how much knowledge people generally have about their previous colonoscopy findings. Some experts suggested including information on histology or the quantity of fecal hemoglobin (f‐Hb), but such details may not be accessible to respondents completing the questionnaire.
11	Previous surgery	3,31	13/17 (76%)	No	15/17 (88%)	Yes	Colectomy (complete or partial) was found highly interesting for CRC risk prediction; however, the experts' opinions were divided on the relevance for other surgeries.
12	History of CRC in the family	4,82	17/17 (100%)	Yes			The experts were divided in their opinions regarding how many degrees of family should be included. Risk of reporting bias/recall bias was identified.
13	Hereditary high‐risk predisposition syndrome in the family	4,71	16/17 (94%)	Yes			Experts agreed that asking about hereditary colorectal cancer syndromes is important. However, several noted that most individuals who *already know* they have Lynch syndrome, familial adenomatous polyposis (FAP), or similar conditions are likely already enrolled in surveillance programs. Therefore, the primary utility of the questionnaire is in identifying those who are *unaware* of their hereditary risk.
14	Fruit and berries	3,82	15/17 (88%)	Yes			From the modified LifeCRC questionnaire
15	Vegetables	4,12	15/17 (88%)	Yes			From the modified LifeCRC questionnaire
16	Dark bread	3,74	13/17 (76%)	No	12/17 (71%)	No	The original questions from the modified LifeCRC questionnaire were revised according to the experts' opinions.
17	Dairy products	3,58	12/17 (71%)	No	12/17 (71%)	No	The original questions from the modified LifeCRC questionnaire were revised according to the experts' opinion.
18	Processed meat	4,41	15/17 (88%)	Yes			The response option to this question from the modified LifeCRC questionnaire was revised according to the experts' opinion.
19	Red meat (livestock meat)	4,03	14/17 (82%)	No	12/17 (71%)	No	This question was not part of the modified LifeCRC questionnaire initially. It was later developed to align with the format of the other food frequency items.
20	Occupational physical activity	4,06	16/17 (94%)	Yes			The item was retrieved from the Saltin‐Grimby Physical Activity Level Scale, which is a validated tool designed to assess individuals' habitual physical activity over a period. It is often used to estimate physical activity levels and their correlation with health outcomes. Comments from the experts revolved around the recall bias and the relevance of a lifelong perspective.
21	Recreational physical activity	4,29	17/17 (100%)	Yes			The item was retrieved from the Saltin‐Grimby Physical Activity Level Scale, which is a validated tool designed to assess individuals' habitual physical activity over a period. It is often used to estimate physical activity levels and their correlation with health outcomes. Comments from the experts revolved around the recall bias and the relevance of a lifelong perspective.
22	Smoking	4,82	17/17 (100%)	Yes			On request from the experts, phrased to adhere to the IASLC language guide. Experts preferred focusing on smoking duration rather than pack‐years.
23	Alcohol consumption	4,71	17/17 (100%)	Yes			Risk for reporting bias was identified. The experts recommended visual aids to improve the accuracy of reporting.

**TABLE 3:** Items excluded in the final version of the *I‐CRC‐RISK Questionnaire*.

Items	Mean relevance for CRC risk prediction (Likert scale1–5)	Comments and considerations
Countries of long‐term residence	2,71 (Round 1)	Identified as interesting for future research, but not rated high enough to be included in the final version. A majority of the experts recommended the item to be excluded. This item was removed after Round 1.
Educational level	2,88 (Round 2)	Educational level was identified as an important factor influencing health literacy and potentially associated with higher adherence to screening programs. While experts acknowledged the relevance of SES as a risk factor, there was some skepticism regarding the use of educational level as an adequate proxy for SES. To broaden the assessment of SES, a new item on household income was introduced. In Round 2, the household income item received a higher rating from the expert group.
For women: Age of menopause	3 (Round 1)	The item was just on the cut‐off mean value, and the group did not appear fully convinced, with six experts recommending its exclusion. In the comments, hormone replacement therapy was highlighted as the most relevant aspect. Therefore, the item was removed after Round 1.
For women: Use of hormonal replacement drugs	3,59 (Round 1)	This item was removed after Round 1 and included in the drug item.
History of cardiovascular disease? (e.g., hypertension, myocardial infarction, angina pectoris, heart failure, stroke)	2,71 (Round 2)	This item was originally suggested by the group during Round 1 but did not reach the cut‐off value. A few experts highlighted hypertension as a potentially interesting risk factor. This item was removed after Round 2.
My dietary pattern is characterized by high intake of fruits, vegetables, legumes, whole grains, lean meats, sea food, and low‐fat dairy products, low intake of processed meat, red meat, saturated fat and sugar‐sweetened beverages and sweets.	In the first draft, the experts were asked to choose between dietary pattern items and food frequency items. The majority preferred food frequency items, and as a result, the dietary pattern items were not included in the final version of the questionnaire. These items were not rated in the first round.	
My dietary pattern is characterized by high intake of red and processed meats, French fries, potatoes, and sources of sugars (e.g., sugar‐sweetened beverages, sweets and dessert foods).		
My dietary pattern is characterized by lacto‐ovo vegetarian food.		
My dietary pattern is characterized mainly by plant‐based food.		
Coffee	2,97 (Round 3)	Viewed by some experts as protective factors; however, the state of evidence was questioned by several experts. The item was deleted in Round 3 due to a low mean rating.
During a typical week, how much time do you spend on vigorous physical activities, i.e., strenuous exercise where you are out of breath, difficult to keep a conversation, such as running, ball sports, gym class?	4,12 (Round 1)	In the first draft, physical activity was presented as three separate items reflecting vigorous physical activity, moderate aerobic activity, and sedentary behavior. Despite the relatively high ratings, the experts expressed concerns about the feasibility and reliability of these items and recommended the use of previously validated questions. As a result, the Saltin‐Grimby Physical Activity Level Scale was introduced in Round 2 and replaced the physical activity items from Round 1.
During a typical week, how much time do you spend on moderate aerobic activities, i.e., you get a little warm and sweaty but can keep a conversation, such as a brisk walk, dancing, pushing a lawn mower?	4 (Round 1)	
During a typical day, how many hours do you spend sitting or lying down (not including night sleep)?	3,88 (Round 1)	

## 4 Discussion

A challenge in CRC risk prediction research is the predictor selection across studies. This paper outlines the development of the *I‐CRC‐RISK Questionnaire* and presents, to our knowledge, the first expert consensus‐based set of self‐reported variables to support next‐generation CRC risk prediction modeling. The *I‐CRC‐RISK Questionnaire* is model‐agnostic, in the sense that it was not developed for a specific model or algorithm and may be applied across different modeling frameworks. The questionnaire should be regarded as a ‘pre‐psychometric’ instrument developed through international expert consensus. It has the potential to guide prospective standardized data collection and transform self‐reported information into a research‐ready format, providing a robust and clinically relevant methodology for data curation. It offers a promising approach to improving the quality and consistency of input data, which remains one of the key limitations affecting the performance of current CRC risk prediction models [15].

The *I‐CRC‐RISK Questionnaire* comprises 23 items. In a Modified Delphi study, including 17 international CRC experts, consensus on the relevance for risk prediction was reached for 19 of these items. Previous CRC risk prediction tools have typically been developed using retrospective cohort data [17]. Commonly included variables are sex, age, family history of CRC, BMI, use of non‐steroidal anti‐inflammatory drugs, physical activity, smoking, and alcohol consumption. Many items in the *I‐CRC‐RISK Questionnaire* have appeared in earlier tools [14, 15, 17], but what distinguishes this questionnaire is the inductive approach used in the modified Delphi study, which produced more nuanced question formulations and response options. For instance, the medication‐use item covers a broader range of drug categories and specifies both frequency and duration. Items covering physical activity, alcohol consumption, and smoking similarly adopt a long‐term perspective to capture habitual patterns rather than momentary behavior. This added level of detail is important, as lifestyle variables predict risk more accurately when collected in greater detail, yet many existing models lack such precision [17]. Particularly noteworthy was the experts' recommendation to include previous participation in CRC screening or colonoscopy, a variable that was not part of the original item pool but emerged through the Delphi process as potentially relevant for CRC risk prediction, which was further supported by recent publications highlighting the importance of screening history in risk stratification [43, 44]. To the best of our knowledge, this is the most comprehensive set of variables to date that aims to capture self‐reported data for CRC risk prediction.

In addition to identifying areas of consensus, the Delphi process highlighted areas of uncertainty regarding several questionnaire items. During the Delphi rounds, the experts provided substantial qualitative feedback on the questionnaire items and their level of detail. Some experts requested more detailed response options, whereas others preferred a simpler format, primarily due to concerns about feasibility. For some retained items, particularly those comprising multiple subcomponents (e.g., medication use), the experts' opinions varied across the individual elements included. These findings should not be interpreted as equal support for all subcomponents but rather as pointing at areas of uncertainty that warrant further empirical evaluation.

Variables represented by items for which consensus on the relevance for risk prediction was not achieved (i.e., *Household Income, Dark Bread, Dairy Products*, and *Red Meat*), or that received ratings in the lower range (e.g., *Waist Circumference, History of Surgery*) need further attention. They should be viewed as provisional and may be considered exploratory until their predictive value has been evaluated in prospective studies. These variables may nevertheless be informative, especially in ML‐based risk prediction models, where complex or only partially understood predictors can be evaluated alongside established risk factors.

Although self‐reported variables are important for CRC risk prediction, they are inherently vulnerable to systematic biases, including recall errors, social desirability bias, and misinterpretation, which may compromise measurement accuracy [45, 46]. In addition, there is often a trade‐off between measurement precision and recall accuracy; while detailed questions may provide a more comprehensive characterization of an exposure, they may also increase respondent burden and the risk of inaccurate reporting [47]. Rigorous psychometric validation is therefore essential, including cooperation with patient representatives.

We recommend further validation work, in accordance with established methodological guidelines, such as the ISPOR Principles of Good Practice, to ensure that these variables reliably and validly capture the intended constructs [48]. The items in the *I‐CRC‐RISK Questionnaire* that received divergent ratings and comments should receive particular attention in future validation studies to determine their contribution to CRC risk prediction. Furthermore, many CRC risk prediction models have been developed in one population but implemented in another, underscoring the importance of validating and adapting such tools to the target population [17]. Prior to large‐scale data collection, the validation process should include translation and cultural adaptation, cognitive interviewing, and pilot testing with representatives of the target population. Comprehensive psychometric evaluation should further assess construct validity and test–retest reliability as well as other relevant psychometric properties. To evaluate the predictive performance of the questionnaire, retrospective registry‐based analyses and prospective cohort studies with registry linkage could be combined, thereby providing complementary evidence on its predictive utility and real‐world applicability.

The *I‐CRC‐RISK Questionnaire* will likely not be a stand‐alone instrument. We envision that self‐reported data collected using a validated version of the *I‐CRC‐RISK Questionnaire* could be combined with registry‐based data, biological markers, and other digital health inputs to support the development of next‐generation CRC risk prediction models. The questionnaire would potentially be used in a screening context and integrated into prediction models used to individualize the screening intervals. A highly accurate and widely implemented risk prediction model has the potential to shift CRC screening from age‐based to risk‐based strategies. Such a shift would primarily benefit screening participants by enabling earlier detection among individuals at higher risk and reducing unnecessary screening among those at lower risk, while also increasing the overall cost‐effectiveness and resource efficiency of screening programs. Although the aim of this questionnaire was to identify key CRC risk predictors regardless of whether they are currently accessible through self‐report or clinical registries, some of the items may be captured more accurately from electronic healthcare records or registries. The optimal mode of implementation, whether completely self‐administered, integrated with registry‐based information, or completed with a healthcare professional, remains to be determined. This decision should be guided by local healthcare contexts, data availability, feasibility considerations, and feedback from intended end users.

The purpose of the Delphi process was not to develop a specific prediction algorithm, but rather to establish a standardized set of predictors for future model development. Such an approach is particularly relevant in the context of ML, where model performance often depends on relevant and well‐defined variables.

Emerging ML approaches show promise in CRC risk prediction and have been claimed to perform better than traditional models [49]. Ensemble ML algorithms can automatically identify the optimal combination of individual learners based on their performance in independent, cross‐validated datasets. Thus, the use of this ML approach, due to being a cross‐validated selector, carries relatively little risk, provided that a wide variety of learners are used in the ensemble implemented with procedures that fairly select the best‐performing model [50]. The ability to find predictive relationships in large and “messy” datasets makes ML a powerful tool for building the next generation of risk prediction instruments. With the growing adoption of ML in medicine, future development, validation, and enhancement of clinical risk‐prediction tools will likely rely on ML models.

Although the Delphi process provides structured guidance for data collection in future risk prediction efforts, ML models may identify additional predictors within datasets that were not previously considered. These models can benefit from, but are not restricted to, the variables included in the questionnaire. By drawing on both evidence‐based and practice‐based expertise, the Delphi process complements risk prediction modeling, which we expect will strengthen the predictive accuracy of future models and enable ML approaches to capture subtle non‐linear relationships between predictors and outcomes that traditional regression methods cannot detect.

Further progress in CRC risk prediction will depend on the availability of high‐quality and comparable data collected across different populations and settings. Therefore, we offer the *I‐CRC‐RISK Questionnaire* as a starting point for collaborative development, and we invite other researchers to test, adapt, and use the questionnaire in future studies to support the advancement of precision screening.

### 4.1 Strengths and Limitations

A strength of the study is the inductive approach applied through the Delphi methodology, allowing for the emergence of variables not previously captured in existing risk assessment tools. The size of the expert group in this study aligns with established recommendations for obtaining relevant feedback from a diverse range of perspectives. The anonymity between the experts and the iterative feedback process helped prevent any single opinion from dominating.

The Delphi method has recognized limitations, including potential selection bias in expert recruitment, limited replicability, the possibility that minority opinions may be overlooked in consensus‐based analyses, and the assumption that panelists possess comparable levels of expertise, which is not always the case [22, 51]. These limitations may influence both the validity and generalizability of the resulting consensus.

To mitigate potential selection bias, expert recruitment was based primarily on recommendations from the IARC (WHO), supplemented by nominations from experts previously approached, rather than relying on our own professional networks. The resulting panel included experts with diverse professional backgrounds and gender representation, which likely broadened the range of perspectives included in the consensus process. In addition, anonymous ratings, predefined consensus criteria, and multiple Delphi rounds with structured feedback were used to reduce the influence of dominant individuals and support independent judgment. Nevertheless, some degree of selection bias cannot be excluded, as expert recruitment inevitably relies on professional networks and may not capture all relevant perspectives.

Another limitation relates to the composition and size of the expert panel. Although the response and retention rates were high throughout the Delphi process, the number of participating experts was relatively modest, although in line with many Delphi studies. While a relatively small panel may limit the breadth of perspectives represented, it also facilitated close engagement with participants, contributing to high response rates and the collection of rich qualitative feedback across rounds.

Also, the panel was predominantly composed of experts from Western countries. Consequently, perspectives from low‐ and middle‐income settings, particularly from South America, Asia, and Africa, may have been underrepresented. Because CRC risk prediction is not uniformly prioritized across healthcare systems worldwide, experts from resource‐limited settings may have brought complementary perspectives reflecting different public health priorities. Furthermore, not all panelists necessarily possessed the same depth of expertise across every questionnaire item, which may have influenced their ratings.

Future validation efforts should therefore incorporate broader geographical representation, including experts and end users from underrepresented regions, and emphasize cross‐cultural adaptation of the questionnaire. This will be particularly important for constructs that are strongly influenced by cultural and socioeconomic contexts, such as dietary habits, physical activity, and income levels.

## 5 Conclusion

In conclusion, we present the *I‐CRC‐RISK Questionnaire*, a core set of self‐reported variables developed through expert consensus. The questionnaire provides a structured foundation for prospective data collection and may support future risk prediction efforts, whether based on traditional prediction models or ML approaches. However, the *I‐CRC‐RISK Questionnaire* should be regarded as a preliminary instrument requiring comprehensive psychometric validation, assessment of predictive performance, and cross‐cultural adaptation before implementation in CRC risk prediction or screening programs can be considered. We invite other researchers to use, refine, and evaluate the questionnaire to advance the field of precision screening.

## Author Contributions

**Hanna Dubois:** conceptualization, funding acquisition, data curation, formal analysis, methodology, writing – original draft, writing – review and editing, investigation. **Eva Torbjörnsson:** conceptualization, writing – original draft, formal analysis, methodology, writing – review and editing. **Jessica Norrbom:** conceptualization, writing – original draft, methodology, writing – review and editing, formal analysis. **Alan Hubbard:** writing – review and editing, methodology. **Mirna Enskär:** conceptualization, writing – original draft, methodology, writing – review and editing, formal analysis. **Rachael V. Phillips:** methodology, writing – review and editing. **Mark van der Laan:** writing – review and editing, methodology. **Johannes Blom:** methodology, writing – review and editing, conceptualization, funding acquisition, writing – original draft, formal analysis, data curation, investigation.

## Funding

This work was supported by Cancerfonden (21 1389 Pj, 22 0599 FE (Blom), 25 4029 FE (Dubois)). Region Stockholm (FoUI‐961412 (Blom)), and Vetenskapsrådet (2021‐03139 (Blom)).

## Conflicts of Interest

The authors declare no conflicts of interest.

## Supporting information


**Supporting Information S1:** The full anonymized dataset from the three Delphi rounds.

**Supporting Information S2:** The international expert group and their credentials.

## Data Availability

The anonymized dataset supporting the findings of this study is provided in the Supplementary S1 accompanying this article.
