# Supplementary material for: Core Self‐Reported Variables for Next‐Generation Colorectal Cancer Risk Prediction: An International Expert Consensus Study
Source: Cancer Med. 2026 Oct 22;15(10):e72348. doi: 10.1002/cam4.72348 (PMC13688420; doi:10.1002/cam4.72348)
Supplement: Supplementary file 1 [file CAM4-15-e72348-s001.docx]

|  | Title/s | Affiliation/s | Gender | City/Country | Expertise |
| --- | --- | --- | --- | --- | --- |
| Anna Martling | Professor, MD | Karolinska Institutet | Female | Stockholm, Sweden | CRC; clinical trials, biomarkers, surgery, radiotherapy |
| Jean-Luc Bulliard | PhD | Centre for Primary Care and Public Health (Unisanté), University of Lausanne | Male | Lausanne/Switzerland | Implementation and evaluation of colorectal cancer screening; risk-stratified CRC screening in a RCT; barriers to participation; production of information material (health literacy); epidemiology and statistics; cancer registry |
| Douglas A Corley | MD, PhD, Chief Research Officer | The Permanente Medical Group; Kaiser Permanente, Northern California | Male | Oakland, California, USA | Research expertise in CRC screening, risk stratification, epidemiology, population screening, familial cancer syndromes |
| Montse Garcia | PhD | 1. Cancer Screening Unit; Catalan Institute of Oncology, Hospitalet del Llobregat, Spain.  2 Early Detection of Cancer Research Group, EPIBELL, Bellvitge Biomedical Research Institute, Hospitalet del Llobregat, Spain. 3. Consortium for Biomedical Research in Epidemiology and Public Health (CIBEResp), Madrid, Spain. | Female | Hospitalet de Llobregat, Spain | Research expertise in CRC screening |
| Iris Lansdorp-Vogelaar | Professor | Erasmus MC University Medical Center Rotterdam | Female | Rotterdam, the Netherlands | Screening evaluation, health economics, epidemiology |
| Douglas J Robertson | MD, MPH, Professor | Geisel School of Medicine at Dartmouth &  The Dartmouth Institute | Male | Hanover, New Hampshire, USA | Expertise in research areas related to this (e.g. diet and incident colorectal polyps, medications and incident polyps etc). |
| Robert A. Smith | Senior Vice President, Early Cancer Detection Science, and Director, American Cancer Society Center for Early Cancer Detection Science | American Cancer Society | Male | Atlanta, Georgia, USA | Led the development of American Cancer Society colorectal cancer screening guidelines since 1997; Health services research in colorectal cancer screening. |
| Robert Steele | Professor, CBE, FMedSci, FRSE | University of Dundee | Male | Dundee, Scotland, UK | Previously Clinical Director of the Scottish Bowel Screening Programme and Chair of UK National Screening Committee |
| David Lieberman | Emeritus Professor of Medicine | Oregon Health and Science University | Male | Portland, Oregon; USA | 35 year history of research related to colorectal cancer screening, surveillance and quality of screening |
| Samar Alhomoud | MD, Professor | King Faisal Specialist Hospital & Research Centre. | Female | Riyadh/Saudi Arabia | Colorectal surgeon/ Research expertise in CRC screening. President of The Saudi Society of Colon & Rectal Surgery |
| Carlo Senore | MD, MSc | Epidemiology and screening Unit - CPO. University Hospital Città della Salute e della Scienza | Male | Turin, Italy | M, epidemiologist. Expertise in the organization and evaluation of screening programs. Research experience in the field of CRC screening. PI of a trial of risk-based CRC screening |
| Kevin Selby | MD, Privat Docent | University Center for Primary Care and Public Health, University of Lausanne | Male | Lausanne, Switzerland | Clinical experience in primary care, expertise in colorectal cancer tests (FIT) and the implementation of risk-based screening. |
| Erin Symonds | Associate Professor | (1) Flinders Medical Centre and (2) Flinders University | Female | Bedford Park, SA, Australia | Research expertise in CRC screening and CRC surveillance. |
| Annika Sjövall | Associate Professor, MD | Karolinska University Hospital | Female | Stockholm, Sweden | Colorectal surgeon, research expertise in CRC epidemiology, treatment, rehabilitation. |
| Edward Giovannucci | MD, ScD, Professor | Harvard University | Male | Boston, MA, USA | Research expertise in CRC epidemiology; general expertise in cancer epidemiology and nutrition, training in medicine, pathology, epidemiology |
| Catherine Dubé | Associate professor, MD, FRCPC, MSc epidemiol | University of Ottawa, The Ottawa Hospital, The Ottawa Hospital research institute | Female | Ottawa, Canada | Research expertise in colorectal cancer screening tests, organized colorectal cancer screening, post polypectomy surveillance; Clinical lead of the province of Ontario's organized colorectal cancer screening program, gastroenterologist |
| Jill Tinmouth | MD, PhD, Professor | Sunnybrook Health Sciences Centre; University of Toronto | Female | Toronto, Canada | My research focusses on screening for colorectal cancer as well as colonoscopy quality improvement and health service utilization |
